# PF^2^ *fit*: Polar Fast Fourier Matched Alignment of Atomistic Structures with 3D Electron Microscopy Maps

**DOI:** 10.1371/journal.pcbi.1004289

**Published:** 2015-10-15

**Authors:** Radhakrishna Bettadapura, Muhibur Rasheed, Antje Vollrath, Chandrajit Bajaj

**Affiliations:** 1 Radhakrishna Bettadapura Computational Visualization Center/Department of Mechanical Engineering, University of Texas at Austin, Austin, Texas, United States of America; 2 Muhibur Rasheed Computational Visualization Center/Department of Mechanical Engineering, University of Texas at Austin, Austin, Texas, United States of America; 3 Antje Vollrath Institut Computational Mathematics, Technische Universität Braunschweig, Braunschweig, Germany; 4 Chandrajit Bajaj Computational Visualization Center/Institute of Computational Engineering & Sciences/Department of Computer Science, University of Texas at Austin, Austin, Texas, United States of America; University of Heidelberg, GERMANY

## Abstract

There continue to be increasing occurrences of both atomistic structure models in the PDB (possibly reconstructed from X-ray diffraction or NMR data), and 3D reconstructed cryo-electron microscopy (3D EM) maps (albeit at coarser resolution) of the same or homologous molecule or molecular assembly, deposited in the EMDB. To obtain the best possible structural model of the molecule at the best achievable resolution, and without any missing gaps, one typically aligns (match and fits) the atomistic structure model with the 3D EM map. We discuss a new algorithm and generalized framework, named PF^2^ *fit* (Polar Fast Fourier Fitting) for the best possible structural alignment of atomistic structures with 3D EM. While PF^2^ *fit* enables only a rigid, six dimensional (6D) alignment method, it augments prior work on 6D X-ray structure and 3D EM alignment in multiple ways:

*Scoring*. PF^2^ *fit* includes a new scoring scheme that, in addition to rewarding overlaps between the volumes occupied by the atomistic structure and 3D EM map, rewards overlaps between the volumes complementary to them. We quantitatively demonstrate how this new complementary scoring scheme improves upon existing approaches. PF^2^ *fit* also includes two scoring functions, the non-uniform exterior penalty and the skeleton-secondary structure score, and implements the scattering potential score as an alternative to traditional Gaussian blurring.

*Search*. PF^2^ *fit* utilizes a fast polar Fourier search scheme, whose main advantage is the ability to search over *uniformly* and *adaptively* sampled subsets of the space of rigid-body motions. PF^2^ *fit* also implements a new reranking search and scoring methodology that considerably improves alignment metrics in results obtained from the initial search.

## Author Summary

In recent years, advances in cryo-electron microscopy (cryoEM) and three-dimensional (3D) image reconstruction have made it possible to determine the structures of molecular complexes, to sub-nanometer resolutions. These reconstructed 3D cryoEM maps provide a unique challenge, since the resolutions are often sufficient to resolve a subset of the secondary structural features (e.g., long *α*-helices), but not high enough to unambiguously identify others (e.g., short *α*-helices, *β*-sheets, *α*-helix pitch, or overall connectivity of proteins). Various types of hierarchical structure refinement models of the data, including the atomistic model match and fitting, which is the subject of this paper are crucial in helping derive a more complete understanding of the structure and function relationships of biological complexes. Our protocol offers several advantages over existing fitting techniques. We introduce three new scoring terms to evaluate the quality of fitting, and the optimization of these functions lead to better predictions than existing tools like ADP-EM and Colores. Furthermore, we have adopted a non-uniform FFT-based search which is not only faster than regular FFT, but it also enables one to selectively perform more refined searches in localized regions, which is specially useful when fitting a small molecular component into a larger symmetric or asymmetric macromolecular structure.

This is a *PLOS Computational Biology* Methods paper

## Introduction

Protein structural data is available in primarily two forms. Atomistic scale structures (or atomic structures for short), acquired through X-ray or nuclear magnetic resonance (NMR) imaging, contain information fine enough to localize the position of most, if not all, the atoms of the protein. However these imaging modalities do not allow a complete picture of the protein’s solvent-induced state. Three dimensional (3D) electron microscopy (EM) maps, reconstructed by single particle (SP) or electron tomography (ET), are at a lower resolution but are easier to obtain and probably closer to the functional native state. A relevant problem of computational structural biology is to reconcile these forms of protein structure data, producing a refined protein model that combines the finer resolution information in the former with the native-state information at lower resolution in the latter. Different frameworks or computational pipelines like comparative modeling, e.g. [1–5] and ab initio modeling, e.g. [6], have played an increasingly important role in this kind of structure determination referred to as the fitting problem. The fitting problem can be solved for either rigid-body (6D) or flexible motions (6D rigid body motion + flexible dimensions) of the atomic structure. In this work, we address aspects common to both problems, and demonstrate results here only on rigid-body fitting.

Approaches to the fitting problem begin by defining a score between an orientation of the atomic structure 𝓟 and the 3D EM map 𝓜. A majority of past work uses the cross-correlation score (CCS) between 𝓜 and a synthesized 3D EM map 𝓜_𝓟_ generated from 𝓟. The CCS is widely used because it is intuitive, easy to implement, and amenable to Fast Fourier transform-based correlations, discussed below. Variants of the CCS include the core-weighted or the Laplacian-filtered CCS [1–4, 6, 7] or normalized cross-correlation (NCC) [8, 9]. There have also been a number of other scoring functions. For instance, the external-total ratio (ETR) measures the total number of atoms of 𝓟 outside a given iso-contour of 𝓜 [10], the vector matching score measures the inner product between a set of vectors representing 𝓟 and 𝓜 [11], while in [12] isosurfaces are matched by comparing surface normals.

A recent review of scoring functions for cryo-EM fitting can be found in [13]. All scoring functions depend on representing 𝓟 (respectively 𝓜), in terms that render it mutually intelligible to 𝓜 (respectively 𝓟). The usual choice, and not necessarily the best choice, for the representation involves blurring 𝓟 by placing a Gaussian at each of its atomic centers. We introduce two representations, termed non-uniform inclusion potential and scattering potential, and show that the scattering potential results in better prediction accuracy. We discuss the details of the terms in the next section and perform a comparative analysis in the Results section.

Once a scoring function is chosen, an algorithm searches for its optima over the space of rigid-body transformations of the protein. Hereafter, we refer to this space as the motion group SE(3). Search algorithms can be usefully distinguished by whether they find local or global extrema of the scoring function. Local optimization is typically synonymous with a variant of steepest ascent [10, 14], although more powerful techniques such as Powell optimization [15] and quadratic programming [5] have also been used. In global optimization, the contest is between Monte Carlo- and Fast Fourier Transform (FFT)-based algorithms. Monte Carlo-based fitting algorithms [4, 16, 17] are able to step past local optima on their way to a close-to-optimal solution; they are easy to implement and widely documented in the literature. Exhaustive or Fourier-based approaches exploit the fact that it is beneficial if the computation of the objective function can be done relatively fast. Fourier-based, deterministic approaches [3, 7, 18–20] guarantee that the found solution is within a user defined error margin of the optimum. Thus they offer a compelling trade-off between accuracy and computation time especially when combined with parallelization techniques or other hardware specific speed-ups, e.g. [9].

We adopt a variant of FFT, the non-uniform SO(3) Fourier transform (NFSOFT) [21] which not only provides better asymptotic computational complexity, but also is specially suited for better sampling of SE(3) and adaptive local searches.

An important aspect of the search procedure is a suitable sampling of the motion group SE(3). Usually the product property SE(3) = ℝ^3^ × SO(3) is exploited for these samplings, where SO(3) denotes the group of three-dimensional rotations, (cf. [22]). Crucial to sampling on SE(3) is sampling of the rotational subgroup SO(3). There are several existing techniques that, given an angular sampling criterion, provide a set of samples that are uniform with respect to accepted metrics of uniformity [23–25].

The paper [7] discusses fast rotational matching, i.e., it omits the translational part of the matching procedure which we incorporate. Hence, the series expansion of the scoring functions used in their work is different, as it uses spherical harmonics but not Laguerre polynomials for the radial part of the function. In contrast to that [19] considers rigid-body motion with rotation and translation. They use a ℝ^1^ × 𝕊^2^ × SO(3) parameterization of the motion group that is different from ours. Their affinity functions are expanded on terms of spherical shells of different radii while we use a decomposition directly on ℝ^3^ using radial wavefunctions in addition to spherical harmonics only. In addition to that our fitting algorithm uses adaptive low-discrepancy samplings, cf. [24] that better reflect the underlying geometries of sphere and rotation group.

After a suitable sampling is obtained the essential mathematical tool needed is the fast calculation of the discrete Fourier transform on the rotation group SO(3) to evaluate the correlation integral that is the objective function. There are several methods to efficiently evaluate Fourier transforms specifically on SO(3) [21, 26, 27]. There are also works that tackle Fourier transforms on the entire motion group SE(3), [28, 29]. The use of fast and efficient algorithms to evaluate the Fourier transform on non-uniformly distributed points, cf. NFSOFT [21] is another improvement of our algorithm. See also Section “Rotational and Rigid-body Correlations; Non-Uniform SO(3) Fourier Transforms”.

**Fig 1:**
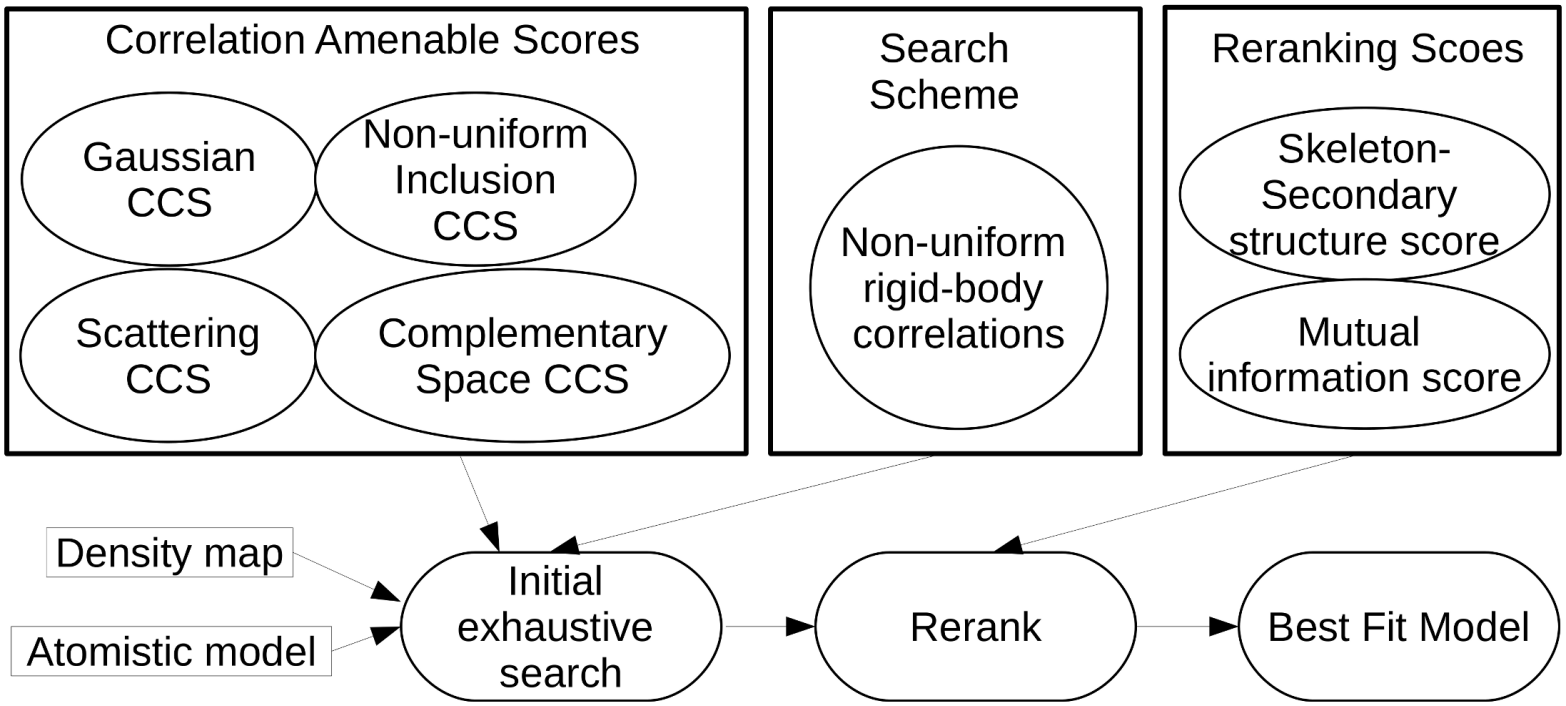
Control flow. A typical control flow of the 3D EM map fitting algorithm developed in this work. The first step of a fitting procedure is the inital exhaustive search. Here one needs to define suitable scoring functions that are amenable for fast correlation computation via the chosen search scheme. Here, we are using FFT-based algorithms for the fast computation of non-uniform rigid-body correlations. The scoring functions may account for various structural aspects such as scattering potential or pockets in the molecular surface. The exhaustive search is followed by an information driven reranking scheme which among others might include the mutual information score or skeleton-secondary structure score. The final output of the procedure will be the best fit between the atomic structure and the 3D EM map.

A schematic overview our algorithm package PF^2^ *fit* which solves the 3D EM map rigid fitting problem, is shown in Fig 1. It introduces the following innovations, each of which lead to improvements over the current state of the art in terms of accuracy and speed.

1. *New FFT-amenable complementary scoring scheme*. The complementary scoring scheme rewards overlaps between the volumes occupied by 𝓟 and 𝓜 as well as overlaps between the volumes *complementary* to 𝓟 and 𝓜. In this context, we introduce two scoring functions: the non-uniform inclusion potential and the complementary space score, both of which are computed on non-uniform grids. We also implement the scattering potential as an alternative to classical Gaussian blurring. The new scoring functions compare favorably to Gaussian-blur-based scoring across a variety of resolutions, in the presence and absence of noise. In particular, our FFT-amenable scoring functions result in lower RMSD than existing ones across a range of resolutions for synthesized density map fitting, and result in lower ETRs for microscope acquired density map fitting, also across a range of resolutions.
2. *Uniform and focused sampling and search with non-uniform FFT*. All prior techniques *require* an equispaced/uniform angular grid for rotational search, a property that results in a highly non-uniform search of the space of rotations SO(3) which is likely to miss important regions of motions while oversampling others. By contrast, *uniform* sampling the space of rotations SO(3), requires non-uniform angular grids (cf. [24]) which is only amenable to a non-uniform, SO(3)-FFT-based search algorithm.
  Furthermore, since our non-uniform FFT framework does not require uniformity of the translational and rotational grids, it enables *focused* searches in both translational and rotational space, thus combining the advantages of local and global fitting schemes.
3. *Information driven rerank scheme*. Finally, to improve the accuracy of our fitting predictions, we rerank results from the search stage with respect to a scoring function based on matching the skeleton of 𝓜 with the secondary structural elements of 𝓟. In the reranking stage, we also include the well-known mutual information score [30].
  Our reranking stage improves the rank of fitting poses obtained in the initial search stage at resolutions < 10Å. We expect the reranking stage to become more effective as more EM maps between 3 and 8Å are isolated.

We should also mention that, due to the improved sampling of SO(3), the time taken by PF^2^ *fit* for an average fitting exercise is comparable to most rival fitting schemes, taking 2–3 mins on an quad-core computer per fitting procedure. In particular, non-uniform inclusion potential takes advantage of the non-uniform search scheme to provide even faster (1.3 mins) runtimes with reasonably accurate estimates of the fitting pose while guaranteeing an exhaustive sampling of the space of available motions. Also, leveraging the focused search capability, PF^2^ *fit* can be applied to a vast range of problem types, from subunit-subunit, to subunit-assembly, to multiple subunit fitting. We have extensively compared PF^2^Fit to ADP-EM (Ref. [3]) in the experiments.

Executable programs as well as the source code for the entire software package PF^2^ *fit* and each of its components libraries are available to all academic users for free through our website. We made the sampling of SO(3) and SE(3), and the non-uniform FFT search libraries separately available so that users can adapt and modify all or some of them independently.

## Materials and Methods

A typical fitting procedure starts with two inputs: an atomic structure 𝓟 and a 3D EM map 𝓜, normally at different resolutions. Let *A* : ℝ^3^ ↦ ℂ and *B* : ℝ^3^ ↦ ℂ be such scalar-valued functions derived from 𝓟 and 𝓜 respectively. Once *A*(**x**) and *B*(**x**): are defined, the best fit of the two molecules is obtained by maximizing the unnormalized cross-correlation score

$$\begin{array}{c} CCS\left(A,B\right)=\int_{{\mathbb{R}}^{3}}A\left(Rx+t\right)B\left(x\right)dx, \end{array}$$ (1)

where (**R**, **t**) is a rigid-body motion, i.e., a three-dimensional rotation ***R*** followed by a three-dimensional translation **t**. Applying the rigid-body motion which produces the maximum score will lead to the best fit.

In this paper, we shall refer to *A*(***x***) and *B*(***x***): as affinity functions. Our fitting procedure is divided into two main stages (cf. Fig 1): the exhaustive FFT-based search, and the reranking. We discuss each of these stages, their affinity functions and their advantages below. Detailed comparison and empirical results are presented in the Results section.

### Non-uniform FFT-amenable affinity functions

**Fig 2:**
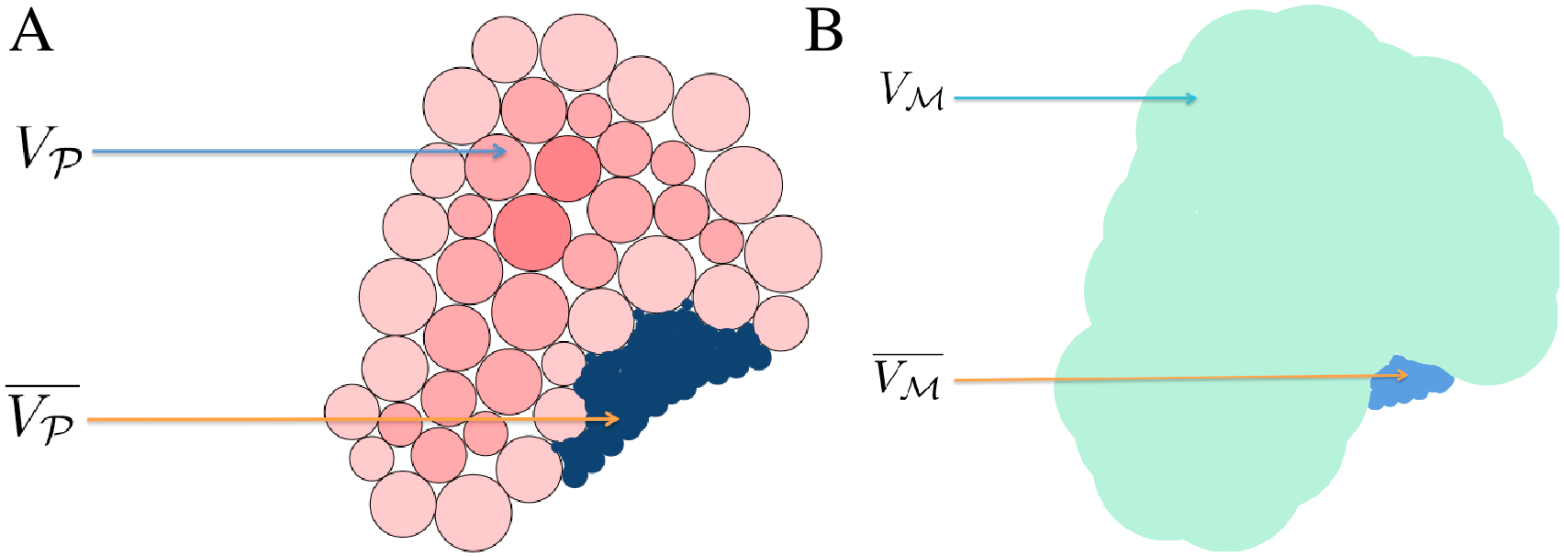
Schematic of representations used in our algorithms. (A) PDB schematic, showing the target volume *V*_𝓟_ and the complementary volume $\bar{V_{𝓟}}$. (B) 3D EM map schematic, showing the target volume *V*_𝓜_ and the complementary volume ${\bar{V}}_{𝓜}$. Detailed definitions can be found in the Materials and Methods section.

PF^2^ *fit* provides four choices for defining the affinity functions of *A*(***x***) and *B*(***x***): the Gaussian *A*_gc_ (respectively *B*_gc_); the scattering potential *A*_sc_ (respectively *B*_sc_); the non-uniform inclusion potential *A*_nu_(respectively *B*_nu_); and the complementary (pocket) space potential *A*_cs_(respectively *B*_cs_). The first three are based on the space occupancy of 𝓟 and 𝓜, and fourth is based on the complementary space of 𝓟 and 𝓜, denoted *A*_nu_ and *B*_nu_ respectively. A typical depiction of the space and complementary space can be found in Fig 2. For a description of how a complementary volume is computed, we refer to [31]. We now discuss each of the affinity functions in detail.

#### Non-uniform inclusion potential cross-correlation score (NCCS)

Let 𝓟_*s*_ be a chosen subset of atoms of 𝓟, and let *X*_*s*_ be the union of spheres of the atoms of 𝓟_*s*_. Then

$$\begin{array}{c} A_{\text{nu}}\left(x\right)=\{\begin{array}{cc} 1, & x\in X_{s}\\ 0, & \text{otherwise}. \end{array} \end{array}$$ (2)

Similarly, let *m* ∈ ℝ be a chosen scalar intensity value. Then

$$\begin{array}{c} B_{\text{nu}}\left(x\right)=\{\begin{array}{cc} 1, & 𝓜(x)\geq m\\ 0, & \text{otherwise}. \end{array} \end{array}$$ (3)

For these definitions of *A*_nu_ and *B*_nu_, a reasonable definition for 𝓟_*s*_ is the set of backbone atoms of the 3D EM map, while *m* can be defined, following [13], as that intensity that results in an isocontour enclosing a volume equal to the volume enclosed by the molecular surface of 𝓟. Note that the envelope score in [13] is a uniform-grid-based version of the non-uniform inclusion potential CCS (hereafter the NCCS).

#### Gaussian cross-correlation score (GCCS)

A classical and widely-used way to represent the protein 𝓟 is by Gaussian blurring, in which Gaussians

$$\begin{array}{c} G^{i}\left(x\right)=\exp(\beta (1-\frac{{∥x-x_{i}∥}^{2}}{r_{i}^{2}}))=\exp(\frac{\pi^{2}\ln2}{R^{2}}\left(1-\frac{{∥x-x_{i}∥}^{2}}{r_{i}^{2}}\right)) \end{array}$$ (4)

corresponding to atom centers **x**_*i*_ and radii *r*_*i*_, are summed at each grid point **x**; the parameter *β* > 0 describes the width of the Gaussian at medium height, *R* is the resolution of the target 3D EM map [32], and

$$\begin{array}{c} A_{gc}\left(x\right)=\sum_{i\in \text{atoms}\;\text{of}\;𝓟}G^{i}\left(x\right). \end{array}$$ (5)

A Gaussian blur is thus a representation that reproduces the electrostatic potential of an atom at points very close to it. This formulation indirectly includes atomic masses by modulating the decay of the Gaussian kernel based on the radii of the atoms.

#### Scattering potential cross-correlation score (SCCS)

An elastic scattering model of the electrostatic potential uses five parameters for each atom, thus yielding a more realistic reconstruction of the electrostatic potential. According to the elastic scattering model [33], the potential at each grid point **x** due to an atom at **x**_*i*_ is given by a sum of five Gaussians

$$\begin{array}{c} V_{sc}^{i}\left(x\right)=\frac{16\pi^{\frac{5}{2}}\hbar^{2}}{m_{0}e}\sum_{j=1}^{5}a_{j}b_{j}^{-\frac{3}{2}}\exp(-\frac{4\pi^{2}{∥x-x_{i}∥}^{2}}{b_{j}+R^{2}}) \end{array}$$ (6)

and

$$\begin{array}{c} A_{sc}\left(x\right)=\sum_{i\in \text{atoms}\;\text{of}\;P}V_{sc}^{i}\left(x\right) \end{array}$$ (7)

where 2*π*ℏ = *h* is the Planck constant, *m*_0_ and *e* are respectively the mass of and charge on the electron, *a*_*j*_ and *b*_*j*_ are empirical parameters [34] that depend on the element type of atom *i*, and *R* is the desired resolution of the representation *A* of the atomic structure 𝓟. Note the functions *B*_*gc*_ and *B*_*sc*_ are identical to the input cryo-EM density map 𝓜 or a suitably filtered version of 𝓜.

The scattering potential is well-known [35, 36]; and has been used for fitting of high- resolution structures to cryo-EM maps in conjunction with constrained geometric simulations [37] or molecular dynamics simulations [38]. The primary motivation behind implementing the scattering potential in PF^2^ *fit* is to explore its value as an alternative to Gaussian blurring since EM reconstruction is based on phase shifts and phase contrast caused by the electrostatic potential. As we show in the Results section), there occur cases, in both acquired and synthesized density map fitting, in which the scattering CCS (hereafter the SCCS) performs better than Gaussian CCS (hereafter the GCCS).

#### Complementary space cross-correlation score (CCCS)

Existing work on rigid-body fitting focuses on representing and correlating the volumes *V*_𝓟_ and *V*_𝓜_ occupied by 𝓟 and 𝓜 respectively. We introduce an addition to the fitting score, the complementary space cross-correlation score (CCCS), that uses the volumes complementary to *V*_𝓟_ and *V*_𝓜_ respectively. We define the complementary volumes as follows.

Let *V*_𝓟_ be the primal volume occupied by the Gaussian molecular surface of 𝓟, and *V*_𝓜_ ⊂ *R* ^3^ is the volume occupied by a suitably chosen molecular surface of 𝓜. Then the complementary volumes ${\bar{V}}_{𝓟}\subset {\mathbb{R}}^{3}$ and ${\bar{V}}_{𝓜}$, are extracted from respective pocket functions [39] that use outward and backward propagation from the primal volumes. Note that, we also use pocket and complementary space interchangeably in the rest of the article.

Given these representations, we can assign *A*_pp_ and *B*_pp_ in Eq (1) as follows:

$$\begin{array}{c} A_{\text{cs}}\left(x\right)=\{\begin{array}{cc} \sqrt{-1}, & x\in {\bar{V}}_{𝓟}\\ 0, & \text{otherwise}. \end{array} \end{array}$$ (8)

$$\begin{array}{c} B_{\text{cs}}\left(x\right)=\{\begin{array}{cc} -\sqrt{-1}, & x\in {\bar{V}}_{𝓜}\\ 0, & \text{otherwise}. \end{array} \end{array}$$ (9)

#### Combining the affinity functions

All the affinity functions we introduced will result in a high positive real *CCS* for large overlaps between target-target or complementary-complementary volumes. One can, in principle, combine all the scoring terms by taking a weighted linear combination where the weights can be either user-specified or optimized by well-known machine learning techniques [40–44]. However, we note that the first three affinity functions represent and the same quantity of interest in slightly different ways and a combination of the three may be redundant. The complementary space score, however, captures a different aspect and should be used in conjunction with any of the first three. Hence, in this work, we compare the predictive performace and speed for each of target-target scores independently and in conjunction with the complementary space score.

#### Expected advantages of the non-uniform FFT-amenable affinity functions

Classical, uniform FFT-based approaches require that affinity functions describing atomic structures or features are mapped onto a uniform grid. This mapping results in either (A) a grid-size much smaller than the average distance between atomic centers, and a resulting increase in time spent on redundant or uninteresting points far from the actual centre of the protein, or, (B) a grid-size much larger than the average distance between atomic centers, resulting in the opposite effect.

By contrast, a feature common to all the affinity functions in PF^2^ *fit* is that they are grid-free, i.e., they do not necessarily require affinity functions to be computed on a uniform grid. This not only mitigates the disadvantage above but leads to ability to perform searches focused to a particular region. The Results section details this advantage of PF^2^ *fit*.

### Non-uniform SO(3) FFT-based Search

The second important ingredient of the inital search stage of rigid-body fitting, is the search algorithm PF*corr* (Polar Fast Fourier Correlation), first introduced in [29], to search over the space of rigid-body motions SE(3) of 𝓟. PF*corr* is a family of rigid-body correlation algorithms based on non-uniform SO(3) Fourier Transforms, and it has many favorable attributes relative to classical FFT-based search algorithms, the most salient of which we discuss here.

#### Multi-basis framework

PF*corr* uses a framework in which scalar-valued functions *A* : ℝ^3^ ↦ ℂ are expressed in terms of basis-expansion coefficients $\hat{a}\in \mathbb{C}$. Let **u** = (*θ*, *ϕ*), *θ* ∈ [0, *π*], *ϕ* ∈ [0, 2*π*], and *r* ∈ ℝ^+^. A scalar valued function *A*(*r*, **u**):ℝ^+^ × 𝕊^2^ → ℂ can be expanded as

$$\begin{array}{c} A\left(r,u\right)=\sum_{k=1}^{L}\sum_{l=0}^{k-1}\sum_{m=-l}^{l}{\hat{a}}_{klm}R_{k}^{\ell }\left(r\right)Y_{\ell }^{m}\left(u\right) \end{array}$$ (10)

where $R_{k}^{\ell }(r)$ and $Y_{\ell }^{m}(\text{u})$ are the radial and spherical basis functions respectively, and *L* is a finite expansion degree. We choose weighted Laguerre radial-basis functions for $R_{k}^{\ell }(r)$ (see [29, 45] for the exact form of these functions), whereas $Y_{\ell }^{m}(\text{u})$ are the well-known spherical harmonic functions.

PF*corr* can also discard the radial-basis functions, following [3], and express each spherical slice *A*_*r*_(**u**) in terms of the spherical harmonic basis coefficients $Y_{\ell }^{m}$:

$$\begin{array}{c} A_{r}\left(u\right)=\sum_{l=0}^{L}\sum_{m=-l}^{l}{\hat{a}}_{lm}Y_{\ell }^{m}\left(u\right) \end{array}$$ (11)

PF*corr*, and hence the algorithm package PF^2^ *fit*, thus support a multi-basis framework, in which a user can choose between either of the two most commonly used bases for rotational speedups. While convenient, the multi-basis framework is not central to our search scheme, and in the interests of brevity, we restrict our discussions below to situations in which the more general mixed bases $R_{k}^{\ell }(r)Y_{\ell }^{m}(\text{u})$ are used.

All of our algorithms extend in a straightforward, if non-trivial way, to cases where the radial basis function is absent and Eq (11) instead of Eq (10) is evaluated at a chosen set of fixed radii *r*, as it is the case in [19] and even [7] where Eq (11) is evaluated for only one fixed *r*.

#### Rotational and Rigid-body Correlations; Non-Uniform SO(3) Fourier Transforms

Let *A*(**x**) and *B*(**x**) be two scalar valued functions with basis coefficients ${\hat{a}}_{klm}$ and ${\hat{b}}_{klm}$ respectively. We are interested in the pure rotational correlation

$$\begin{array}{ccc} C(R) & = & \int_{{\mathbb{R}}^{3}}A\left(Rx\right)B\left(x\right)dx\\ & = & \sum_{k\ell mm^{\prime }}{\left(-1\right)}^{m}{\hat{b}}_{k\ell -m}{\left(-1\right)}^{m^{\prime }}\bar{{\hat{a}}_{k\ell -m^{\prime }}}D_{\ell }^{m,m^{\prime }}\left(R\right), \end{array}$$ (12)

and the rigid-body correlation

$$\begin{array}{ccc} C(R,t) & = & \int_{{\mathbb{R}}^{3}}A\left(Rx+t\right)B\left(x\right)dx\\ & = & \sum_{klmn}{\hat{b}}_{k\ell m}D_{\ell }^{n,m}\left(R^{B}\right)\sum_{k^{\prime }\ell^{\prime }m^{\prime }}{\left(-1\right)}^{n}{\hat{a}}_{k^{\prime }\ell^{\prime }m^{\prime }}D_{\ell^{\prime }}^{-n,m^{\prime }}\left(R^{A}\right)T_{k\ell ,k^{\prime }\ell^{\prime }}^{\left|n\right|}\left(z\right), \end{array}$$ (13)

where (**R** ^*A*^,**R** ^*B*^, *z*) is the factorization of the rigid-body transformation (**R**, **t**) into rotations of *A* and *B* and a single translation of *A* along the z-axis [29]. The effect of rotation is described by the Wigner-D functions $D_{\ell }^{m,m^{\prime }}$ that are a set of basis functions for *L* ^2^(SO(3)). The effect of this translation is described by a translation tensor *T*(*z*) with elements $T_{k\ell ,k^{\prime }\ell^{\prime }}^{|n|}(z)$, cf. [46].

This factorization of a motion into five rotational degrees of freedon and one remaining translational degree has been used in the field of protein matching by [19, 47] before. However, here it will be applied in a uniform setting and a fast evaluation algorithm for the first time.

PF*corr* [29] provides a pair of recipes to compute each of the above sums. The technical content of these recipes can be found in our work on non-uniform multi-dimensional correlations [29]. For the purposes of this work, the most relevant fact is its use of the non-uniform fast SO(3) Fourier transform (NFSOFT) [21]:

$$\begin{array}{c} f\left(R_{i}\right)=\sum_{l=0}^{L}\sum_{m=-l}^{l}\sum_{n=-l}^{l}{\hat{f}}_{lmn}D_{l}^{m,n}\left(R_{i}\right),\;i\in \left\{1\ldots N_{R}\right\} \end{array}$$ (14)

where ${\hat{f}}_{lmn}\in \mathbb{C}$ are the input SO(3) Fourier coefficients of *f* ∈ *L* ^2^(SO(3)). The Wigner-D functions $D_{l}^{m,n}$ form a orthogonal basis of *L* ^2^(SO(3)), and *i* indexes *N*_**R**_ non-uniformly spaced z-y-z Euler Angles in SO(3).

This is a significant improvement over existing fitting tools. Due to the limitations of the uniform-FFT techniques that underly them, all current rotationally efficient methods [3, 7, 47] depend on a uniform discretization of Euler angular space. Unfortunately, because the space of Euler angles is a non-linear parametrization of the target space of rotations SO(3), this leads to a highly *non-uniform* set of samples in SO(3).

The expansion degree *L* embodies an aspect of the speed-accuracy tradeoff: higher degrees result in a greater ability to capture shape information in the 3D EM map, while causing an obvious degradation in performance. We find that setting *L* anywhere between 20 and 30 suffices for 3D EM map fitting exercises. The NFSOFT can be used to compute the above sum in 𝓞(*L* ^3^log*L* + *N*_**R**_) steps, in contrast to the generally far slower naive 𝓞(*L* ^3^ *N*_**R**_) approach.

#### Adapting PF*corr* for Fitting

We can see that the above recipes can be conveniently incorporated into a fitting search algorithm. As a preprocessing step, we compute the basis-expansion coefficients ${\hat{a}}_{klm}$ and ${\hat{b}}_{klm}$ of *A*(**x**) and *B*(**x**) respectively. Then based on the two recipes, one can choose either of the following search schemes.

1. *Search choice 1: PF^2^*fit*—SE(3)*: Compute Eq (13) in 𝓞((*L* ^6^ + *L* ^4^ *N*_**R**^*B*^_ + *N*_**R**^*B*^_ *N*_**R**^*A*^_)*T*) steps over appropriate sample sets {**R** ^**A**^}, {**R** ^**B**^}, and {*dz*} where *N*_**R**^*B*^_ and *N*_**R**^*A*^_ are respectively the sizes of the sample sets {**R** ^*A*^} and {**R** ^*B*^}.
2. *Search choice 2: PF^2^*fit* —SO(3)*. For each **t** over a set of samples in ℝ^3^, translate *A* by **t** and recompute ${\hat{b}}_{klm}$; then compute Eq (12) in 𝓞(*L* ^4^) steps over a set of samples in SO(3).

While PF^2^ *fit* —SE(3) can be used along with any sampling technique, we use the sets {**R** ^**A**^}, {**R** ^**B**^}, along with equispaced grid values for {*dz*} for the first fitting stage in order to avoid overseeing important regions of motions while oversampling others. This low dispersion and low discrepancy sampling of SO(3) [24, 29] is highly advantageous in the first stage of fitting as explained in the next paragraph.

PF^2^ *fit* —SO(3) maximizes rotational scanning at the expense of translational scanning, an approach also adopted in [19] and [3]. Note that both these methods use a framework that excludes the radial basis function $R_{k}^{\ell }$, e.g. due to the consideration of star-shaped molecules and discretizations of the radial parts respectively.

#### Expected advantages of the non-uniform SO(3) FFT-based search

Like the NFSOFT, PF^2^ *fit* scales gracefully under non-uniform discretizations of the space of z-y-z Euler Angles. This is a significant improvement over existing fitting tools [3, 7, 47] that depend on a uniform discretization of Euler angular space. Hence, for a given angular step size, these methods will always generate sample sets in SO(3) that examine parts of that space very finely while leaving others undiscovered. By contrast, an advantage of PF^2^ *fit* is its ability to work efficiently with an arbitrary set of samples in Euler angular space, using a sampling technique such as in [24]. This is the primary advantage of PF^2^ *fit* as a search algorithm. The consequences of this advantage extend naturally to all results obtained by PF^2^ *fit*.

### The Reranking Scheme

In the second stage, results obtained in the correlation-amenable search stage are reranked with respect to scores that exhibit the following features: (A) They cannot be expressed in the general form of Eq (1). (B) The information they capture about a particular fitting orientation is additional to, or, ideally, independent of, each of the affinity functions maximized in the search stage.

#### Skeleton-secondary structure score

We introduce a reranking score that depends on the detection of secondary structural features from 𝓜. This has been a vigorous area of research in the past decade; for a recent review, see, for instance [48]. We use the skeletonization technique in [20, 48] to detect secondary structures from 𝓜, and the publicly available Stride [49] to detect the secondary structures of 𝓟. Let 𝓗_𝓜_ and 𝓗_𝓟_ respectively be the set of helices detected from 𝓜 and 𝓟. Each helix consists of an axis **r**, with ‖**r**‖_ℓ_2__ = 1, and a midpoint **p**. Let ${\text{h}}_{i}^{𝓜}$ be a helix in 𝓗_𝓜_, and let ${\text{h}}_{j}^{𝓟}$ be a helix in 𝓗_𝓟_. Let *d*(.,.) be the Euclidean distance function, ⟨.,.⟩ be the dot product, and *w*_1_ ∈ ℝ^−^, *w*_2_ ∈ ℝ^+^ be respectively negative and positive weights. Then the per-helix score and the secondary structural score are respectively given by

$$\begin{array}{c} SSS_{h_{j}^{𝓟}}=\max_{i}w_{1}d\left(p_{i}^{𝓜},p_{j}^{𝓟}\right)+w_{2}\left|\left\langle r_{i}^{𝓜},r_{j}^{𝓟}\right\rangle \right| \end{array}$$ (15)

$$\begin{array}{c} SSS=\sum_{j}SSS_{h_{j}^{P}}. \end{array}$$ (16)

In this work, we set *w*_1_ = −1, *w*_2_ = 1, in which case the theoretical range of the per-helix score $SSS_{{\text{h}}_{j}^{𝓟}}$ is (−∞,1]. The best possible per-helix score corresponds to the situation where the helices are perfectly aligned and have the same mid point, and $SSS_{{\text{h}}_{j}^{𝓟}}=1$. In most practical scenarios, $SSS_{{\text{h}}_{j}^{𝓟}}$ is typically between 0.25 and 0.7.

#### Mutual information score

The second reranking function we use is the mutual information score (see, for instance, [13, 30]), given by

$$\begin{array}{c} MIS=\sum_{x\in B}\sum_{y\in A}p\left(x,y\right)\log(\frac{p(x,y)}{p(x)p(y)}), \end{array}$$ (17)

where *p*(*x*) and *p*(*y*) are the percentage of voxels in *B* and *A* that take on intensities equal to *x* and *y* respectively and *p*(*x*, *y*) is the percentage of voxels in *B* with intensity *x* that are aligned with voxels in *A* with intensity *y*. In PF^2^ *fit*, *A* and *B* correspond respectively to the target volumes *V*_𝓟_ and *V*_𝓜_ respectively, with the former computed by the Gaussian blurring scheme.

#### Expected advantages of the reranking scheme

The reranking stage serves two purposes. The first is to identify spurious results. Any fitting method that depends purely on a set of correlation-amenable affinity functions has the potential to yield high-scoring results that are nevertheless obviously incorrect. The second, related, goal of the reranking stage is to bring the process of rigid-body fitting closer to automation. We see fitting as a single stage in the elucidation of structure from biological data. The elucidation process comprises several data processing stages, and it is critical that the output of each stage is as accurate as it can be. One very popular way to measure the accuracy of a fitting algorithm is to perform a simple visual check; however, it may be time-consuming or otherwise impractical to visually check every single fitting pose generated by an automated fitting algorithm such as the one presented here. In these situations, the reranking procedure will either provide additional guarantees that the top result is in fact the one that fits the best, or will flag results whose scores do not agree about the quality of the fit.

### Experimental Setup

We carried out experiments to compare different scoring and reranking functions implemented in PF^2^ *fit* as well as to compare PF^2^ *fit* with other publicly available fitting software, namely the Colores tool in Situs [18] and ADP_EM [3]. In this section, we describe the benchmark dataset, the experiment protocol and the metric used in measuring and comparing accuracy of fitting. The results and their implications are presented in the next section (Results and Discussion).

#### Dataset

For experiments involving synthesized 3D EM maps, we used a variety of atomic structures from the PDB. Many of these atomic structures overlap with those in the docking benchmark [50]; they were chosen mainly for their diversity in size and shape (average TM-score [51] between the structures is 0.27165, which indicates very low structural similarity). The PDB IDs of the 53 atomic structures we used are: 1QG4a, 1OUNab, 1D6Oa, 1IASa, 2TGT, 1K9Ba, 1MH1, 1HH8a, 1FPZf, 1B39a, 2CGAb, 1EGL, 1AY1hl, 1CMWa, 1BJ1hl, 2VPFgh, 1A6Zab, 1C68ab, 1GJRa, 1CZPa, 1TNDc, 1FQIa, 1FGNlh, 1TFHa, 1HCL, 1DKSa, 1GJRa, 1CZPa, 2CLRde, 1CD8ab, 1FSKbc, 1BV1, 1IJJb, 3DNI, 1BVLba, 3LZT, 9RSAb, 2BNH, 1TRMa, 1ECZab, 1FSKbc, 1BV1, 1E1Na, 1CJEd, 4PEP, 1F32a, 1BDD, 1FC1ab, 1QHDa, 1OELg, 1AONa, 1CTS, 2CTS and 1Q3Qa. We generated synthesized 3D EM maps for each model 𝓟 of this benchmark, by first blurring it at a fixed resolution *R* to produce a synthesized map *B* which mimics an EM; and then a random transformation is applied to the original model 𝓟 to generate 𝓟′. Now, the task is to find the best fit between 𝓟′ and the map *B*.

For acquired 3D EM map experiments, we used a selection of datasets from the CryoEM Challenge [20]. The resolutions for these cryoEM 3D EM maps range between 3.8Å to 20Å and is hence lower than for the synthesized data.

#### Experiments

We performed the following experiments to validate PF^2^ *fit*. Each of the experiments inherently validates the search scheme introduced in this work. Additionally, they validate, compare and highlight aspects of one or more of our affinity functions and scores.

**Fig 3:**
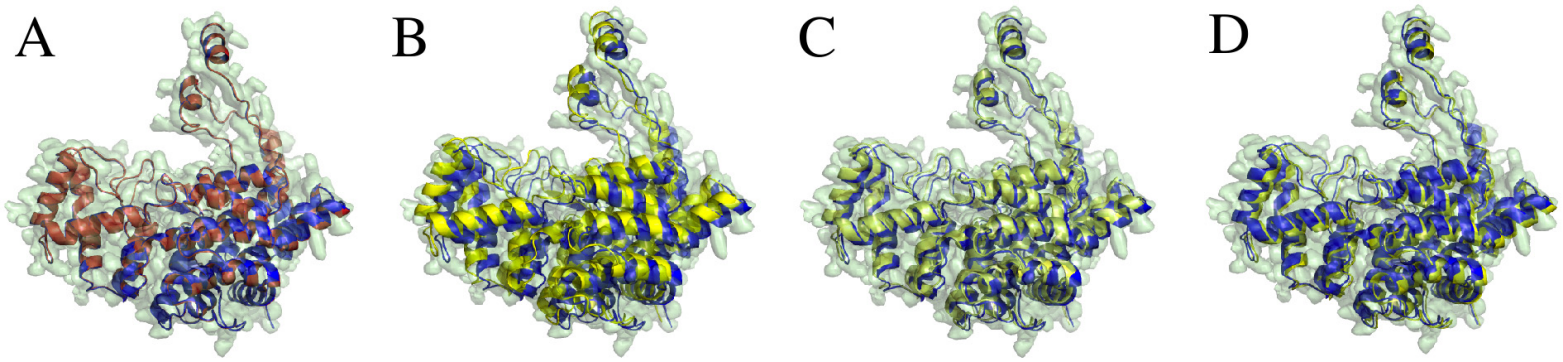
Comparison of PF^2^ *fit* with other software in synthesized EM fitting at 3Å. A molecule is fitted into the synthetically generated EM map *B* with resolution 3Å(transparent green). The top-ranked result 𝓟_1_ (red/yellow) is compared to the original PDB molecule 𝓟 (blue). (A) Top-ranked result using PF^2^ *fit* —SE(3) with 8° uniform rotational sampling and 0.5Å translational step size. RMSD ≈ 0.88Å. (B) Top-ranked result using the Colores package; the ‘nopowell’ option is turned on. RMSD ≈ 3.2Å. (C) Top-ranked result using Colores with default options. RMSD ≈ 2.3Å. The fitted PDB 𝓟_1_ is in yellow. (D) Top-ranked result using the ADP_EM package, with bandwidth *L* = 25. RMSD ≈ 0.94Å.

**Fig 4:**
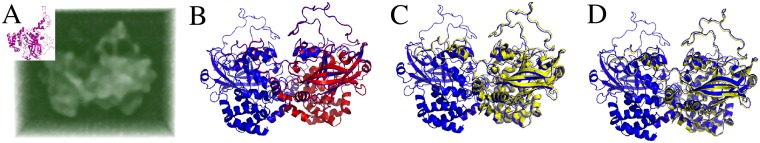
Comparison of PF^2^ *fit* with other software in synthesized EM fitting at 10Å. (A) The synthetically generated 3D EM map is a Gaussian blurred version of the PDB 7CAT (chains A and B), with resolution *R* = 10Å, and random noise added to obtain a signal-to-noise ratio of unity. The PDB 𝓟 (inset) is chain B of the same protein. The top-ranked result 𝓟_1_ (red/yellow) is compared to the original PDB molecule 𝓟 (blue). (B) Top-ranked result using PF^2^ *fit* —SE(3) with 8° uniform rotational sampling and 0.5Å translational step size has RMSD = 0.73Å. (C) Top-ranked result using Colores with default options has RMSD = 1.096Å. (D) Top-ranked result using the ADP_EM package, with bandwidth *L* = 25 has RMSD = 0.814Å.

- *Validating different scoring terms using synthesized 3D EM map and comparison with other software*. We applied our PF^2^ *fit*—SE(3) and PF^2^ *fit*—SO(3) algorithms using each of our target-target scores GCCS, NCCS and SCCS, independently, to predict the orientation of 𝓟′ that produces a good fit to *B*. Examples of this experiment are visualized in Figs 3 and 4.
  We repeated the experiments with the complementary scoring term (CCCS) added in and compared our obtained results to the results reported in [3] on similar experiments carried out with colores [18] and ADP_EM [3]. We also compared the performance of PF^2^ *fit* with other software in fitting electron microscope acquired data for subunit-subunit and subunit-assembly cases. Details are presented in the Discussions Section. See also [20].
- *Analyzing resolution robustness of scoring and search using synthesized 3D EM maps*. In reality, the EM maps come in many different resolutions. To verify that our scoring models and search scheme preserves their applicability across a wide range of resolutions, while making fitting predictions with high accuracies, we progressively coarsened the resolution *R* of the target blurred 3D EM map *B*, and repeated the above experiments for each level of coarsening. Finally, the experiments were repeated with random Gaussian additive noise added to *B*.
- *Analyzing the effect of reranking using synthesized 3D EM map*. To measure the efficacy of the reranking metrics, we examined the 53 PDBs in our synthesized dataset with resolutions between 5 and 15Å, with a step size of two, thus conducting 318 fitting experiments.
- *Analyzing the effect of various samplings using synthesized 3D EM map*. To evaluate the speed-vs-accuracy tradeoff for PF^2^ *fit*, we applied PF^2^ *fit* on the synthesized dataset while varying the density of the sampling of SO(3) used in the search. For each of the structures, we used 1854, 4392, 8580, 14868, 29025, 68760, and 232020 samples on SO(3) corresponding to, respectively, rotational separations of 20, 15, 12, 10, 8, 6 and 4 degrees between samples. Additionally for the NCCS, we ran the same experiments while varying the expansion degree (L).
- *Performance on acquired 3D EM map Fitting*. We applied PF^2^ *fit* to acquired cryoEM data, which is more challenging than synthesized map fitting since it may contain non-random noise, differences in conformations of the molecule, and possibly more than one molecule in a complex. We performed three types of fitting with acquired EM maps. First, we used PF^2^ *fit* —SE(3) and—SO(3) to fit PDB subunits to subunits segmented from the 3D EM map (subunit-subunit). Segmentation was performed using the methods reported in [52, 53]. Second, we used PF^2^ *fit* —SE(3) to fit a single PDB subunit into a larger 3D EM map (subunit-assembly). And third, we used PF^2^ *fit* —SE(3) to fit multiple PDB subunits into a larger 3D EM map.

#### Validation Metrics

For the experiments involving synthesized maps, the true position or true fitting is simply the original position of the PDB model 𝓟. After a randomly oriented copy 𝓟′ of the model is fitted using PF^2^ *fit*, it produces a new position and orientation, 𝓟′′. For perfectly accurate fitting, 𝓟′′ should perfectly coincide with 𝓟. We simply compute the root mean square distance (RMSD) based on the positions of the atoms in 𝓟′′ and 𝓟, as a measure of the accuracy such that lower RMSD indicates a better fitting prediction.

For both synthesized and acquired EM maps, we additionally use the external total ratio (ETR) as a metric of fitting quality. ETR is the ratio of the number of atoms outside a suitably picked isocontour of the 3D EM map to the total number of atoms in the PDB. We term this score the external-total ratio (ETR) [20]. ETR is a very subjective measure as it depends on the choice of the isocontour and hence should only be used when a better metric (e.g. RMSD) cannot be computed.

Finally, as a measure of the confidence on the prediction, we use Z-score. The Z-score [54] of a fitting result is given by $z=\frac{x-\mu }{\sigma }$, where *x* is the score of the fitting result, and *μ* and *σ* are respectively the average and the standard deviation of the population. The Z-score measures the degree to which a scoring function can discriminate between two different candidate solutions, with higher scores indicating better discriminatory ability.

## Results and Discussion

In this section, we discuss the relative performance of the different Cross Correlation Scoring (CCS) terms introduced in PF^2^ *fit*, in terms of the results obtained from the experiments on both synthesized and electron microscope acquired data and compare the performance of PF^2^ *fit* with Colores and ADP_EM [3]. We also discuss the effect of reranking shemes, skeleton-secondary structure score (SSS) and the mutual information score (MIS). Finally, we discuss some unique advantages offered by PF^2^ *fit*.

### The scattering potential CCS is a valuable alternative to the Gaussian CCS

Gaussian blurring have usually been the method of choice for fitting software. In this paper, we introduced the scattering potential which provides a better model of the electron density than Gaussian. In our experiments on both synthesized and acquired EM data, we found that SCCS is a valuable alternative to the GCCS, one whose performance is similar and stable across a range of resolutions.

#### On synthetized EM data

**Fig 5:**
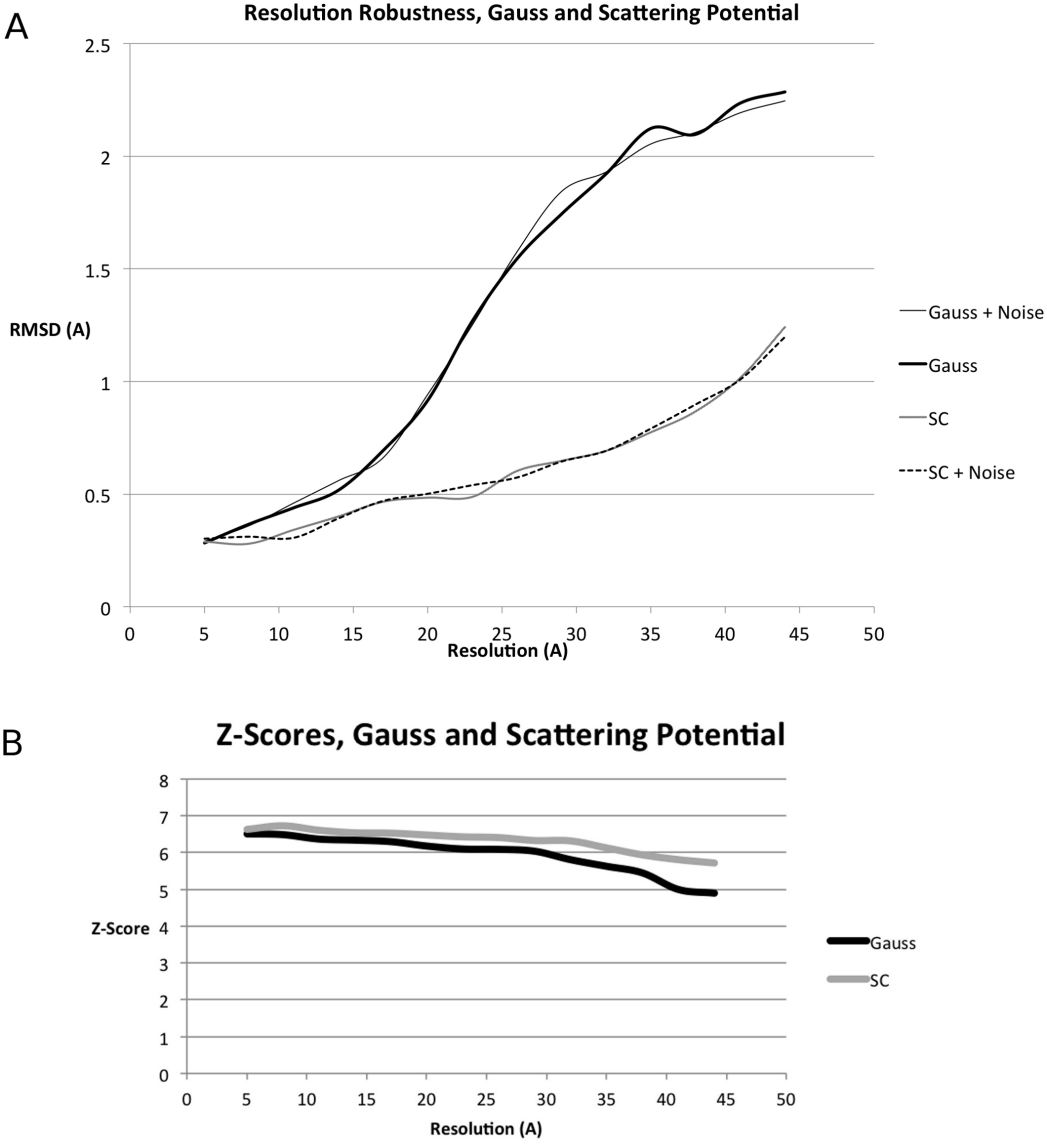
Resolution robustness and comparison of scattering potential (SCCS) and Gaussian (GCCS) scores for synthesized data. We plot the RMSD of the top-ranked result as a function of the resolution of the EM map used for the fit. See the section titled “Dataset” for a list of PDBs used in this experiment. (A) Average resolution-dependent RMSD of the top-ranked result returned by PF^2^ *fit* —SE(3) in the absence and presence of noise for the GCCS and the SCCS. (B) Average Z-Score for the ten top results in the absence of noise. Z-Scores in the presence of noise follow the same trend.

**Fig 6:**
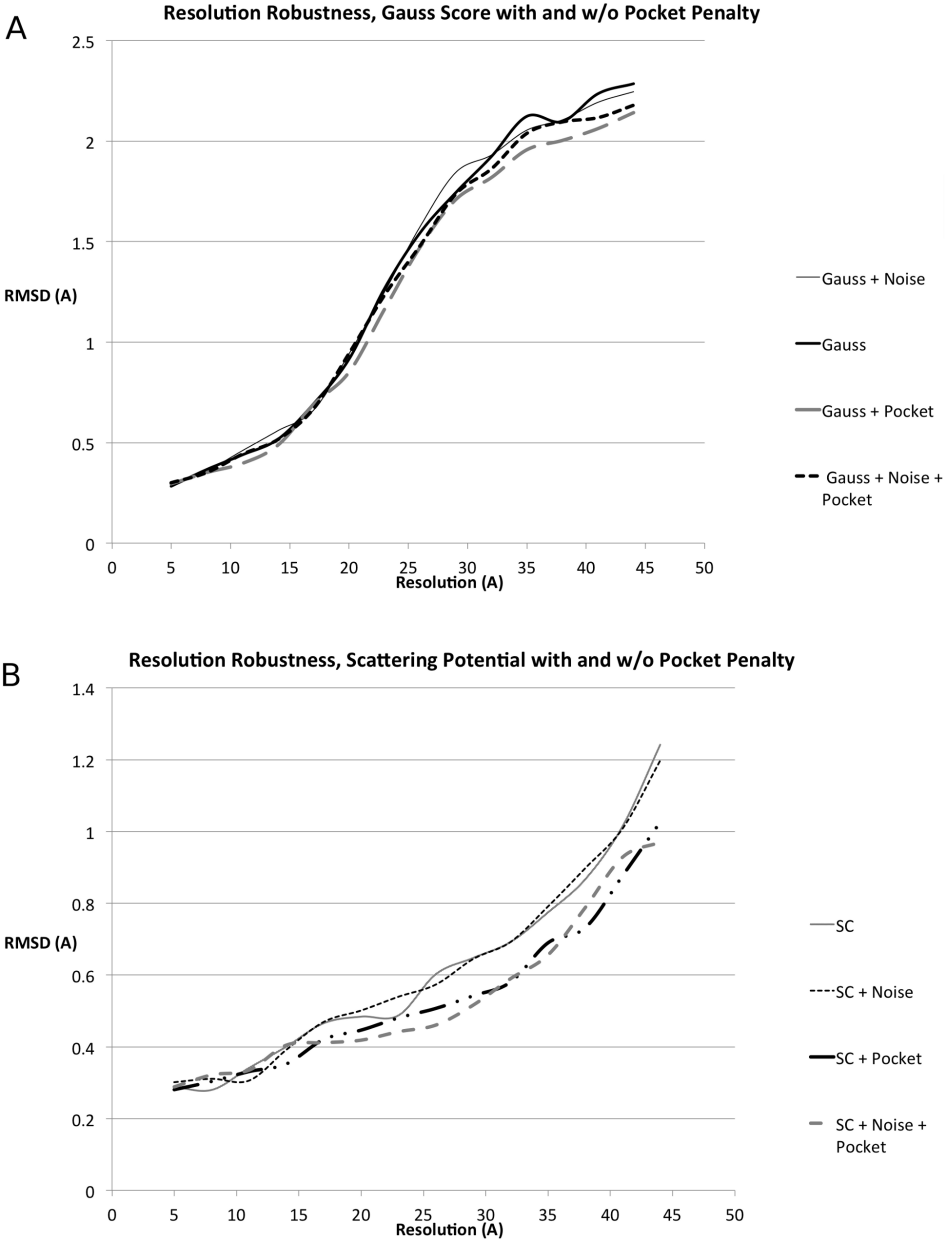
Effect of complementary space scoring for synthesized data. Using the complementary space scores from (A) Eq (8) and (B) Eq (9), with *w*_comp_ = 1, *w*_target_ = 1 we plot the RMSD as a function of the resolution of the EM map. See the section titled “Dataset” for a list of PDBs used in this experiment.

We compare the GCCS with the SCCS in Table 1, Figs 5 and 6. When compared to the GCCS, the SCCS produced results with lower RMSD for the same resolution, both in the presence and the absence of noise, cf. Figs 5 and 6. However, the average rank of the of the best RMSD result might be lower for SCCS than GCCS as can be seen in Table 1.

**Table 1:** Average rank, rounded to the nearest integer, of best RMSD result returned by PF^2^ *fit* —SE(3) in the initial search stage for synthetic maps at different resolutions. The figure in brackets in the second and third columns denotes the rank in the presence of noise at SNR = 1. See the section on “Datasets” for a list of PDBs used in this experiment. Note that even if the rank of the best RMSD is lower for SCCS in some cases, the actual RMSDs are generally lower, cf. Figs 5 and 6.

Resolution (Å)	Rank—GCCS	Rank—SCCS
5	1 (1)	1 (1)
9	1 (1)	1 (1)
13	1 (1)	1 (2)
17	2 (2)	2 (3)
21	1 (3)	2 (5)
27	1 (3)	3 (5)
31	2 (3)	3 (5)
35	2 (5)	4 (7)
39	2 (7)	5 (7)
43	2 (7)	5 (7)
47	2 (8)	6 (7)

Another illuminating trend is the slope of each of the curves in Fig 5, which reveals that results returned by the GCCS degrade more sharply than those from the SCCS. Note that both the GCCS and the SCCS yielded better RMSDs on average than ADP_EM (see [3], Fig 1), yielding on average lower RMSD results at the same synthesized EM resolution.

Table 1 gives the the average rank of the best RMSD result returned by PF^2^ *fit* —SE(3).

#### On acquired EM data from the cryoEM challenge

We also compared results obtained from acquired cryo-EM 3D EM map fitting (Experiment 3) using both the GCCS and SCCS. In this experiment, the RMSD cannot be measured, as there is no atomic structure corresponding to 𝓜. Instead, we use the number of atoms excluded outside a given iso-contoured molecular surface to compare the performances of the rival CC scores. The results, in Table 2, show that for fitting with acquired 3D EM maps, the SC yielded on average results that exclude 2–4 fewer residues than the GCCS. This is in keeping with the expectation that the SCCS is closer to acquired 3D EM map reconstructions than the GCCS, and is thus able to yield a better correlation in situations where such 3D EM maps are involved.

**Table 2:** Results of applying PF^2^ *fit* —SE(3) on a selection of datasets from the cryoEM modeling challenge (Experiment 3) using both the GCCS and SCCS. An error measure similar to ETR is provided as the number of residues excluded outside a given iso-contoured molecular surface. The SCCS yielded on average results that exclude 2–4 fewer residues than the GCCS.

Type of Experiment	Data	GCCS	SCCS
subunit-subunit	GroEL _(𝓟 = 1AONb, 𝓜 = EMD 1461 @7.7 Å)_	4	0
subunit-subunit	GroEL _(𝓟 = 1OELg, 𝓜 = EMD 5001 @4.2 Å)_	3	0
subunit-assembly	GroEL _(𝓟 = 1WE3b, 𝓜 = EMD 1180 @7.7 Å)_	3	0
subunit-subunit	mm-cpn _(𝓟 = 1Q3Qb, 𝓜 = EMD 5137 @ 4.3 Å)_	2	0
subunit-subunit	Rotavirus _(𝓟 = 1QHDa, 𝓜 = EMD 1461 @ 3.8 Å)_	3	0
multiple subunit-assembly	SIV _(𝓟 = 3DNO, 𝓜 = EMD 5020 @ 20 Å)_	17	10

Put together, these observations imply that the SCCS is an effective alternative to the GCCS in microscope acquired density-map fitting scenarios, since it is a more realistic representation of a 3D EM map. This conclusion applies not just to the problem of rigid-body fitting, considered in this work, but to the harder problem of flexible fitting, where it is critical that the representation of 𝓟 be as close to the target 3D EM map as possible. We note that there is a tiny overhead in computing the SCCS relative to the GCCS due to the former being a sum of five Gaussians.

Fig 5 suggests that for lower resolutions (< 20Å), it might be sensible to use more resolution robust scoring functions like the cross-correlation score normalized with respect to mean value and standard deviation as proposed in [9]. These normalized scoring function could be used in a straightforward manner in our PF^2^ *fit* software either as a preprocessing step for the EM maps or as an additional FFT-amenable scoring function.

### Complementary space cross-correlation score improves predictions

The addition of the complementary space cross-correlation score (CCCS), or the pocket score, (Fig 6) to the scoring function resulted in tangible improvements to the quality of the obtained fit across the range of resolutions. This was observed with the GCCS (Eq (5)), as well as the SCCS, and both in the presence and absence of noise. The improvement in the quality of the results obtained is most dramatic at values of *R* beyond 15Å.

### Low Discrepancy Sampling Results in Better Speed-Accuracy Trade-off

Fitting is essentially an optimization problem in a high dimensional configuration space. In PF^2^ *fit*, and any other existing methods, the configuration space is discretized to a small number of discrete samples where the scoring terms are evaluated and the maximum/minimum is reported. Now, let 𝓕 be the scoring term and 𝓒 be the configuration space, from which *N* discrete samples are taken. Then if the true maximum value is m*(𝓕) = *max*_*x*∈𝓒_𝓕(*X*), and the sampled maximum value is *m* ^𝓢^(𝓕) = *max*_*x*∈𝓢_𝓕(*X*)- then it is guaranteed that m*(𝓕)−*m* ^𝓢^(𝓕) ≤ *ω*_𝓒_(𝓕, *d*_*N*_) (see Theorem 6.4 in [55]), where *d*_*N*_ is the dispersion of the samples in 𝓒, and *ω* is a measure of the continuity of 𝓕. So, for a given 𝓕 and 𝓒, the error is directly correlated with dispersion of the *N* discrete samples.

In PF^2^ *fit*, we use the low-discrepancy and low-dispersion sampling scheme for SO(3) space described in [24]. By contrast, existing fitting software [3, 7, 47], due to their use of uniform-FFT, requires uniformity in the parameters used to represent the orientations in SO(3), for example uniform sampling of *θ*, *ϕ* and *ψ* Euler angles or icosahedral vertices on the sphere as in [3]. But such uniform sampling of the parameters might lead to *non-uniform* set of samples in SO(3), leaving large gaps (high-dispersion) in some places, showing high discrepancy or not sufficiently reflecting the topological structure of the underlying domain, cf. [24]. Since, PF^2^ *fit* uses a non-uniform polar FFT (NFSOFT [21]), it is able to handle non-uniformity in the Euler angles (which leads to uniformity, in low-discrepancy sense, in SO(3)). As a result PF^2^ *fit* can achieve high accuracies even with very limited number of samples.

**Fig 7:**
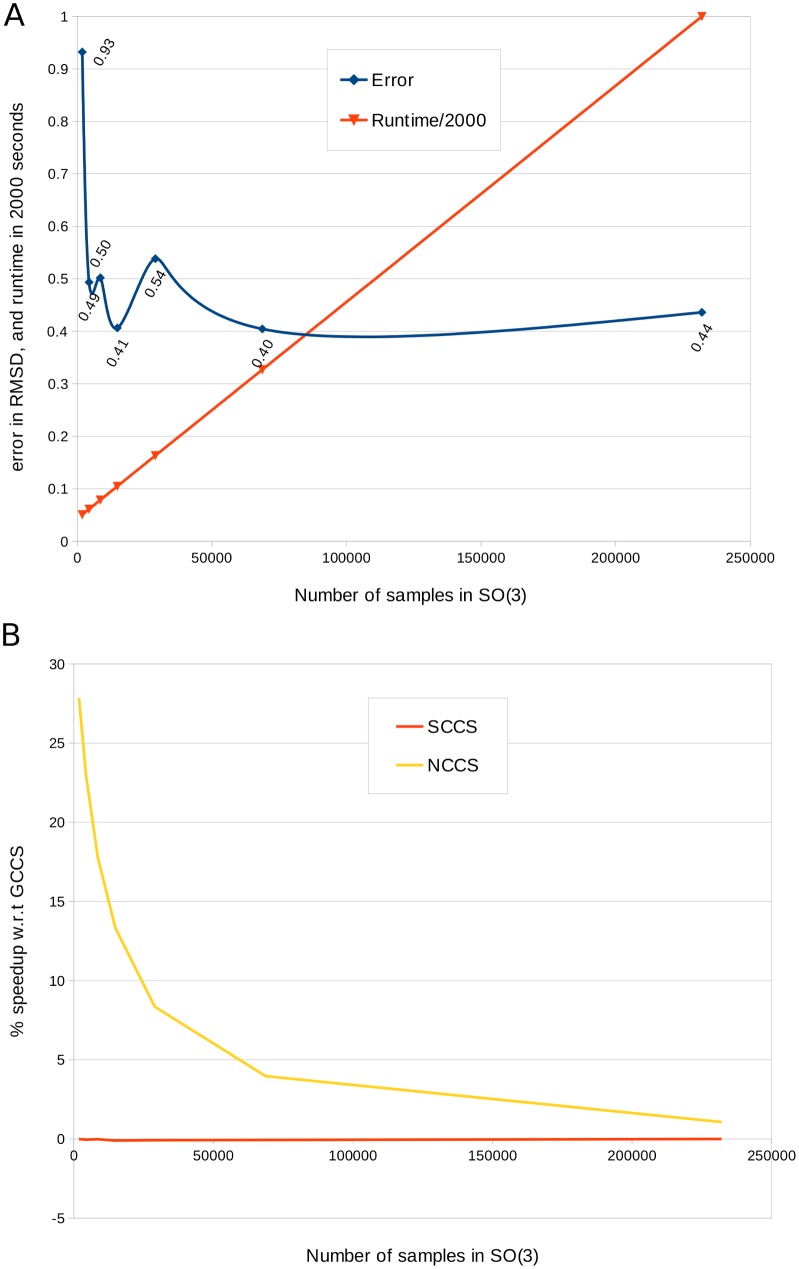
Speed-accuracy trade-offs in PF^2^ *fit*. (A) The plot displays the average runtime (divided by 2000) using the GCCS scoring term, and the corresponding error (in RMSD) when PF^2^ *fit* is applied on the synthesized EM dataset. Notice that the runtime increases linearly with the number of samples in SO(3), but the average error is quite steady between 0.4 to 0.5Å except for the case when only 2000 samples were used. We believe that such robustness stems from the low discrepancy of the sampling. (B) We compared the average speeds of PF^2^ *fit* on the synthesized EM dataset with GCCS, SCCS and NCCS using the same expansion degree (L = 20). The plot shows that NCCS is faster than GCCS, specially when fewer samples are used. On the other hand SCCS is marginally slower (around 0.1%) than GCCS.

In Fig 7(A) we report the results of applying PF^2^ *fit* on the synthesized EM dataset, with different number of low-discrepancy samples in SO(3). Notice that the results are stable with only around 5k samples (corresponding to about 15 degree separation in SO(3)) which runs in around 100 seconds in a single threaded execution. The Non-uniform inclusion potential cross-correlation score (NCCS) highlights another advantage of non-uniform FFT. NCCS incurs much smaller overhead than GCCS or SCCS, and the advantage becomes more pronounced when the overall runtime is smaller (see Fig 7(B)). For example, at 5k samples, the NCCS is about 20% faster than GCCS. Further advantages of NCCS, in terms of its speed-accuracy tradeoff is discussed in a later section.

### The performance of reranking increases with resolution

We discuss the result of applying two separate reranking schemes, the skeleton-secondary structure score (SSS) and the mutual information score (MIS) in terms of improving the ranks of the predictions from PF^2^ *fit*’s FFT-amenable initial scoring phase which have low RMSD.

#### On synthetized EM data

We tested the described set of 53 models in our synthesized EM dataset for 6 different resolutions and then counted the instances in which the reranking replaced the top-ranked result of the initial stage with a previously lower ranked one, that has a better RMSD and is hence a better fit.

In Table 3, we report the average rank of the lowest RMSD prediction, after applying the reranker in absence and presence of noise dependend on the resolution. We see that MIS is more resolution robust than the SSS and both perform better at higher resolutions.

**Table 3:** Average rank of best RMSD result returned by PF^2^ *fit*—SE(3) after reranking. In the initial stage GCCS was used. The figures in brackets denote the rank in the presence of noise at SNR = 1. We see a strong decrease in rank for the skeleton-secondary structure score with and without noise while the mutual information score remains predictable across the range of resolutions. See the section on “Datasets” for a list of PDBs used to generate the synthetic maps used in this experiment. Note that even if the ranks of the best RMSD solution, on average across all experments, show no improvement over Table 1 (mostly because GCCS already does an excellent job of ranking them)- the ranks actually improved for several of the experiments (73/318 for MIS, and 5/318 for MIS). Please see Section ‘The performance of reranking increases with resolution’ for details.

Resolution (Å)	Rank SSS	Rank MIS
5	2.91 (7.72)	1.04 (1.67)
7	2.95 (7.86)	1.05 (1.62)
9	3.79 (8.86)	1.04 (1.74)
11	8.93 (13.15)	1.06 (1.88)
13	9.97 (25.89)	1.06 (1.75)
15	15.89 (48.17)	1.08 (1.83)

The MIS replaced the top-ranked result of the initial stage with one that has a better RMSD 73 times, predominantly at resolutions below 10Å. These cases occured for all the PDBs listed in the section on datasets, except 1BVLba, 1AY1hl and 2BNH.

On the other hand, we notice that the usefulness of the SSS degraded sharply with decreasing resolution. There were only five cases out of the 318 experiments in which the SSS replaced the top-ranked result with a result that has better RMSD; all of these cases occured at resolutions < 10Å, and for the PDBs 1FSKbc, 1BVLba, 1BDD, 2CTS and 1OELg. However, we expect the SSS to become a more effective gauge of fitting quality as the quality of density maps obtained from cryo-EM increases, and more density maps at resolutions between 3–5 Å are isolated.

#### On acquired EM data from the cryoEM challenge

For finer resolution 3D EM maps, such the one corresponding to GroEL at 4.4Å, mm-cpn at 4.3Å, Rotavirus at 3.8Å, or GroEL at 7.7Å, SSS correlates with the MIS, as well as the FFT-amenable fitting metrics, about the quality of the fitted result, generating scores that range between 0.3 and 0.8 for the top ten fitting results. However, for 3D EM maps such as those of GroEL at 11Å or SIV at 20Å it diverges sharply from the MIS and the other measures introduced in this work, and yields results that are clearly, i.e., visually, incorrect, due simply to the quality of skeleton obtained.

We expect the SSS to become a more effective gauge of fitting quality as the quality of 3D EM maps obtained from cryo-EM increases, and more 3D EM maps at resolutions between 4–8Å are isolated.

### PF^2^ *fit* compares favourably with other publicly-available software

We tested PF^2^ *fit* against the publicly available ADP_EM and Colores. Though these three programms have correlation-based fitting schemes, the exact formulation of the affinity functions, and just as importantly the sampling scheme are not the same. For instance the translational step of PF^2^ *fit* is not directly comparable to the translational step of Colores. Also, the angular sampling density as well as the expansion degree affect the outcome. So, it is almost impossible to come up with parameters for each software which would result in the same measure of dispersion in the samples. Hence, we fixed the parameters of PF^2^ *fit* such that its runtime is similar to those of ADP_EM and Colores in their default settings, and then compared the results.

#### Accuracy on synthetized data

In Fig 5 we report the average RMSD of the top-ranked fitting predictions made by PF^2^ *fit* over the set of 53 PDBs mentioned in the Validation and dataset section. As the PDBs were blurred, the accuracy (RMSD) of the prediction gradually got worse, as expected. We compared these to the plot in Fig 1 of [3], where performance of ADP_EM and Colores is reported in the same manner (i.e. RMSD vs. resolution) for a smaller dataset. According to the data reported in [3], the average RMSD of three variants of ADP_EM and Colores+Powell is above 1Å when the blurring is greater than 20Å, which is very similar to PF^2^ *fit* using GCCS. However, PF^2^ *fit* with SCCS achieved average RMSDs which are less than 1Å, even when the model is blurred upto 40Å, and is hence much more robust for fitting to low resolution maps. The lowest blurring resolution reported in [3] is 10Å. At that resolution, the approximate average errors for Colores+Powell, the best variant of ADP_EM, PF^2^ *fit* with GCCS and PF^2^ *fit* with SCCS are respectively 0.25, 0.7, 0.4 and 0.3 respectively, indicating that PF^2^ *fit* performs comparatively well when better maps are available as well.

For PF^2^ *fit*, the top-ranking or second-ranking pose was observed to be usually the one with the least RMSD (Fig 5). This feature should be common to all global-optimisation-based fitting routines in which atomic structures are rigidly fit to blurred versions of themselves. However, we observed in our experiments that while the top ranked fitting result in Colores is usually also the one with the least RMSD, the ones ranked 2–10 have RMSDs that can range anywhere between 1.1 and 10 times the RMSD of the top-ranked result. We surmise that this spuriousness is an artifact of its Powell maximization step.

An example of how PF^2^ *fit* performs relative to Colores and ADP_EM [3] can be seen in Fig 3. This is an instance of Experiment 1 applied to a single chain atomic structure. The fitted result using PF^2^ *fit* results in an RMSD of 0.88Å, the least of the three programs, while Colores and ADP_EM respectively return 3.2 and 0.94Å.

Another example, a variant of Experiment 1, can be seen in Fig 4. Here a random rigid-body transformation (**R**, **t**) is applied to a single chain 𝓟_*a*_ of a two-chain atomic structure 𝓟_*ab*_. 𝓟_*a*_ is then fitted to a synthesized EM density map generated from 𝓟_*ab*_ to generate ${𝓟}_{a}^{fit}$, using one of PF^2^ *fit*, Colores, or ADP_EM. The RMSD between ${𝓟}_{a}^{fit}$ and 𝓟_*ab*_ is then measured. Fig 4 shows that PF^2^ *fit* obtains an RMSD of 0.73Å, while ADP_EM and Colores obtain an RMSD, respectively, of 0.814Å and 1.096Å.

All in all, our experiments showed that PF^2^ *fit* can be considered as a viable alternate with features and tradeoffs that complements those available in ADP_EM and Colores.

#### Performance on acquired EM data from the cryoEM challenge

**Fig 8:**
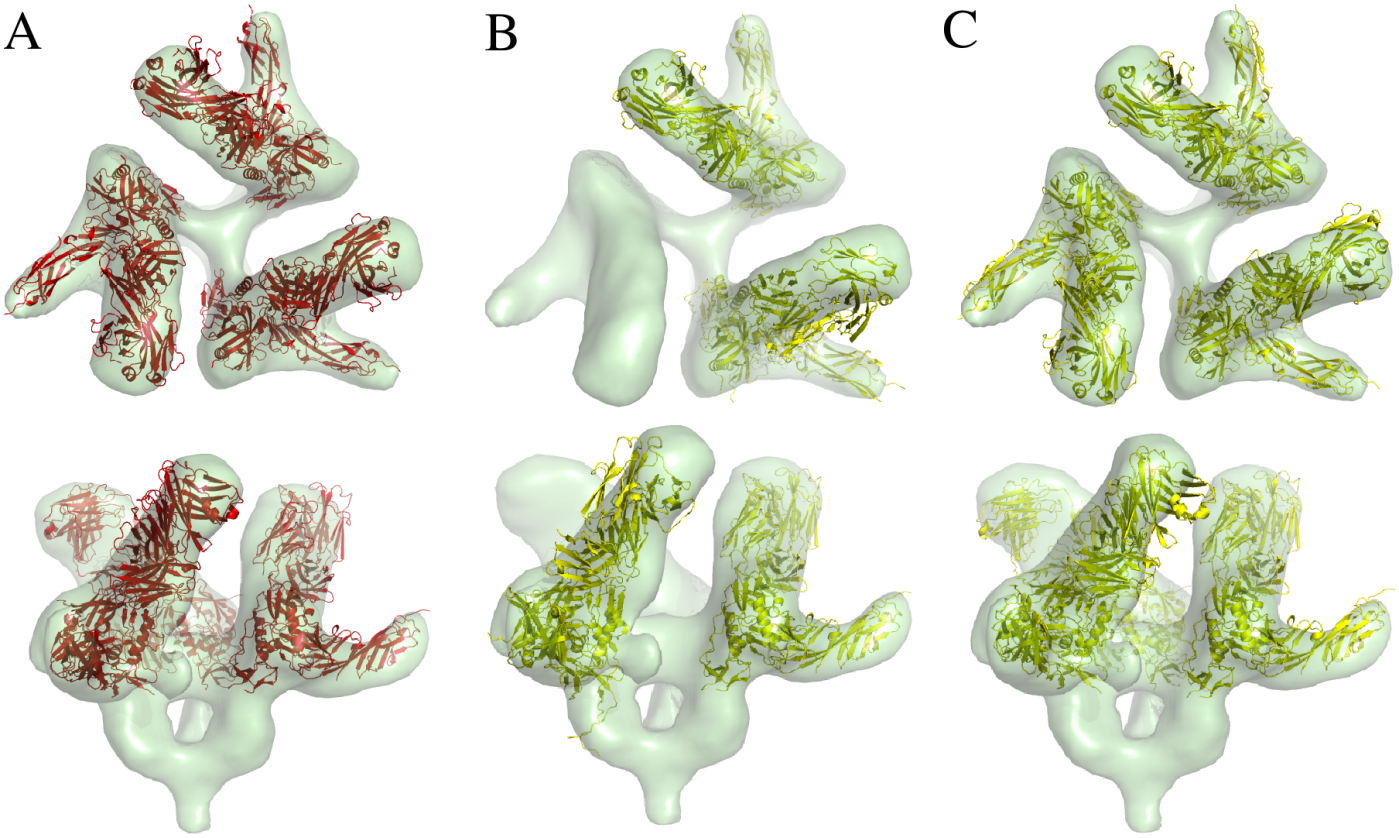
Comparison of PF^2^ *fit* with other software in subunit-assembly fitting. Fitting the PDB molecule 𝓟 (1GC1) to the EM map 𝓜 of SIV 20Å(EMD5020), using the GCCS. Two different views of the molecules are given: (A) Results from PF^2^ *fit*. The ETR is 0.03. (B) Results from colores with default options. The ETR is 0.1. (C) Results from ADP_EM with *L* = 25. The ETR is 0.08.

An example of acquired EM data subunit-assembly fitting is provided in Fig 8. Here a single subunit of an atomic structure is fit to a larger density map which contains three repetitions of the subunit. An ideal fitting routine should be able to rank each of the symmetric positions of the fitted atomic structure above all other hypothetical positions. While PF^2^ *fit* and ADP_EM achieve this, Colores is unable to find one of the three symmetric positions in its top ten results. Additionally, PF^2^ *fit* attains the lowest ETR of the three fitting routines. The ETRs for PF^2^ *fit*, Colores, and ADP_EM are, respectively, 0.03, 0.1, and 0.8.

#### Timing

As previously mentioned, the parameters in PF^2^ *fit* were set up to yield comparable runtimes to ADP_EM [3] and Colores. An average PF^2^ *fit* fitting exercise with *L* = 25 and angular sampling of 10° per rotational degree of freedom takes is around twice as much time as ADP_EM [3] with default settings, and less time than Colores (even when Powell optimization is not done). For example, fitting the Beef Liver Catalase (PDBID: 7CAT) on a single-threaded 2.5GhZ processor with 8 GB main memory takes about 2.5 minutes for PF^2^ *fit*, 65 seconds for [3] (with the same value of *L*), and about 3.5 minutes for Colores. These reported times are averaged over 25 executions of each. Note that, for PF^2^ *fit* most of the performance overhead is due to the non-uniform nature of the search algorithm, and in particular the NFFT.

### Subunit-assembly benefits from the unique focusing ability of PF^2^ *fit*

**Fig 9:**
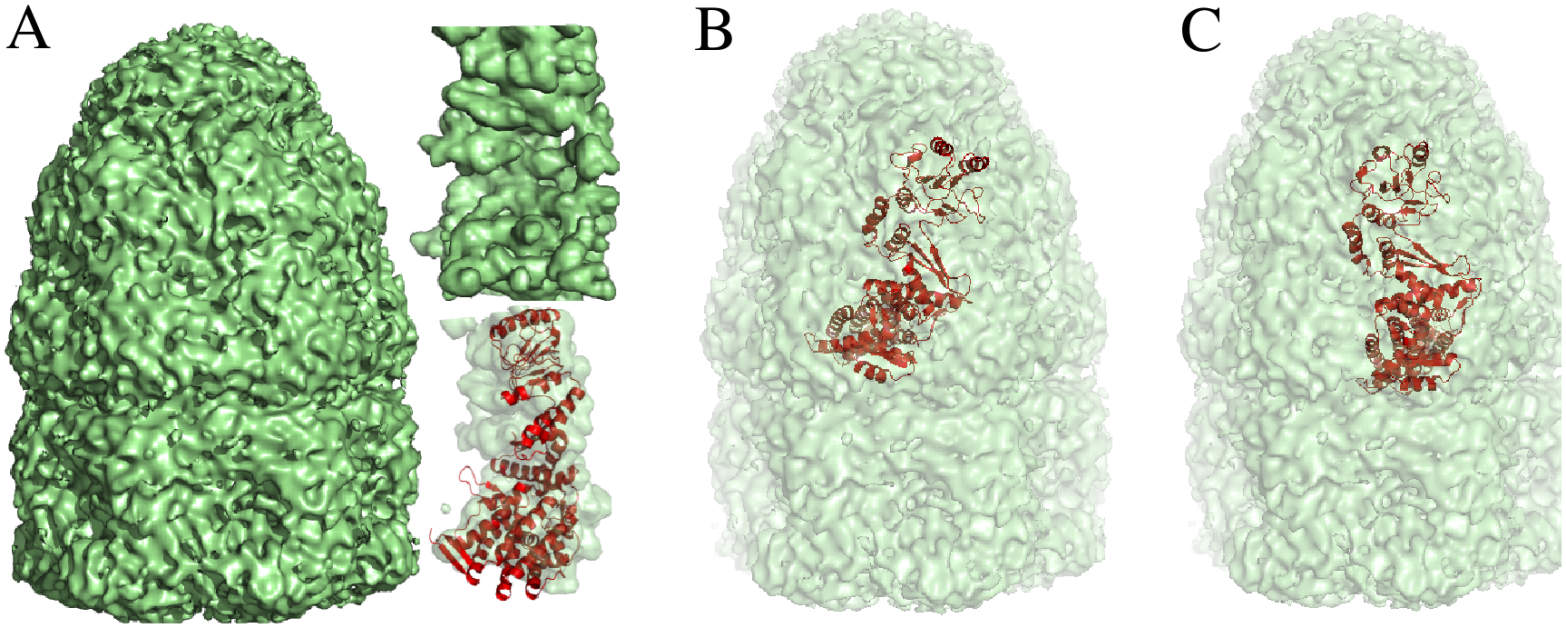
Fitting the PDB molecule 𝓟 (1AONb) to the GroEL 3D EM map 𝓜 (EMD 1461) at 7.7Å. (A) Full 3D EM map 𝓜 with segmented subunit 𝓜_*s*_ (inset, top). The molecule 𝓟 is fitted into 𝓜_*s*_ using PF^2^ *fit* (inset, bottom). (B) Initial guess for rigid-body fit into 𝓜. (C) PF^2^ *fit* generates translational samples local to the initial guess to find the depicted correct result. Correctness is measured by deviation from the rigid-body fit in (A). The result has an RMSD of 0.3Å from the fitting result in (A) and is ranked at number four in a run of PF^2^ *fit* with angular resolution of 10°.

Suppose an experimenter wants to refine the cryo-EM map of GroEL at 7.7Å (𝓜 = EMD 1180, 192 × 192 × 192 voxels) by fitting a single subunit of GroEL (𝓟 = 1AONa) into it. This is the subunit-assembly problem, in which the translational uncertainty is roughly twice the size of 𝓟, whereas the rotational uncertainty is the range of rotations from 0 to 2*π*. One way to effect the refinement would be to segment from 𝓜 a 3D EM subunit of GroEL 𝓜_*s*_, to which 𝓟 could be fitted using PF^2^ *fit* —SE(3) (Fig 9A).

If a good segmentation is unavailable, a software like Chimera [10] could be used to refine an approximate placement. Another option is to use rigid-body fitting with Colores. Chimera surveys a fixed number (=50) distinct poses in its gradient-descent-based optimisation scheme, and the fit obtained is only locally optimal. On the other hand, Colores uses a uniform Cartesian grid with a default translational step equal to the voxel spacing of the map. With a default angular fineness of about 30° on a cubical Euler Angle grid, this results in 64^3^ = 262144 translational samples and 864 rotational samples. Of these, several positions are redundant, as they lie outside the region of the initial guess. In general, there is no way in Colores to confine the translational search to a given region.

A third option is to use PF^2^ *fit*. The focusing property of PF^2^ *fit* mitigates both the above disadvantages (Fig 9B-9C). Since the GroEL assembly is symmetric, the experimenter could place the subunit approximately within the 3D EM map (Fig 9B), and then instruct PF^2^ *fit* —SE(3) to do a comprehensive search in the local region around the 3D EM map. In such an experiment, translations are completely disabled, and the rotational search space is uniformly sampled. Using the uniform SO(3) sampling technique in Mitchell [24] yields 14868 samples at 10° angular step. The result in Fig 9C is obtained.

Using PF^2^ *fit* in such scenarios has the following advantages. First, the comprehensive search in the local region essentially guarantees that PF^2^ *fit* —SE(3), unlike iterative gradient-descent-based optimization techniques, is not sensitive to an initial guess. Second, unlike global search routines, PF^2^ *fit* does not generate spurious rigid-body fits in regions that are spatially distant from the optimal fit. Third, the time-taken for the experiment is proportional to the number of local samples rather than for the (much larger) entire search space. PF^2^ *fit* thus combines the merits of local and global search paradigms in its focused search.

Note that many rotational-FFT-based schemes, e.g. [3] share the focusing property; however, since these techniques use a cubic Euler Angle grid, they do not ensure that the space of rotations is uniformly sampled.

### The non-uniform inclusion potential (NCCS) is highly efficient

**Fig 10:**
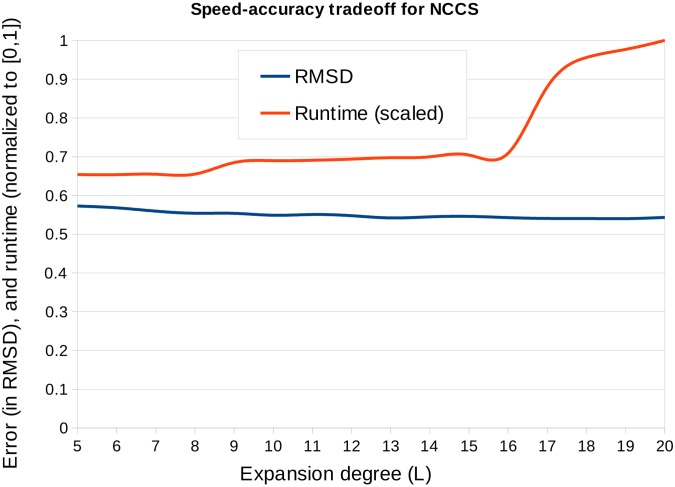
Speed-accuracy trade-offs for NCCS. NCCS is computed on a non-uniform grid based on the atom positions. If the grid is sparse, then it is expected that a lower degree expansion of the spherical basis functions would sufficiently represent it. We applied NCCS with the expansion degree (L) varied between 5 to 20, on the synthesized EM dataset (blurring to 12A resolution) and using 30k samples in SO(3) space. The plot shows that the error decreases and runtime increases with L. However, the change is runtime is more pronounced than the change in error, for example, the runtime is 35% faster for L = 5 while the error is only 5% more than that of L = 20.

The NCCS is a non-uniform-grid-based version of the envelope score in Vasishtan and Topf [13]. Along with PF^2^ *fit*, the non-uniformity inherent to the inclusion potential enables a very high speed search of the space of rigid-body motions SE(3) available to 𝓟. We explain this by first noting that since the quantity of information in *A*_*nu*_(**x**), cf. Eq (2), is exactly equal to the number of atoms in 𝓟_*s*_, a relatively low degree *L* in Eq (10) suffices to represent it. In general, while the GCCS and SCCS each demand a degree at least equal to *L* = 20, with best results for *L* ≥ 25, the NCCS requires only a degree *L* = 5 (see Fig 10).

By itself, however, this property is of limited use. In the uniform-FFT frameworks used in either [47] or [3], the expansion degree is keyed directly to the coarseness of rotational sampling, because the underlying FFT grids are only as fine as the expansion degree allows them to be. Using a degree *L* = 5 in either of these approaches would mean conducting a rigid-body search over an angular grid with separation 360/(2 × 5) = 36°, an unacceptably coarse value for most rigid-body fitting exercises. By contrast, PF^2^ *fit*, functioning as it does through the *non-uniform* SO(3) Fourier transform, enables an arbitrarily fine scan of the space of rigid-body motions at *any expansion degree*.

These advantages mean that the NCCS can play a central role in rigid-body fitting. If a coarse estimate of a fitted position of 𝓟 with respect to 𝓜 is desired, then a low expansion degree version of the NCCS can be used, whereas a more accurate estimate can be found using the SCCS at *L* ≥ 20. The typical time taken for a subunit-subunit fitting exercise on a single-threaded Macbook Pro at 2.5 GhZ with 8GB RAM is about 1.3 minutes.

### Concluding remarks

The results of this paper has contributed to existing methods and software on rigid-body fitting. In particular:

- *Cross Correlation scoring functions*. We have introduced the non-uniform inclusion potential CCS in Eqs (2), (3). This score has been shown to be preferable to standard fitting metrics in terms of speed (cf. Fig 10). We have also introduced the concept of complementary space matching, and introduced the complementary space scoring function (CCCS). The addition of the CCCS results in significant improvements in the prediction accuracy across a range of resolutions, regardless of the target-target scoring function used. Finally, we have compared the scattering potential (SCCS) with the typically used Gaussian potential (GCCS), finding that it performs favourably in our application compared to the latter in both synthesized and microscope acquired density map fitting scenarios and hence provides a valuable alternative.
- *Search scheme*. We have introduced a search scheme that is resolution-robust, capable of local fitting, and able to quickly and comprehensively survey the space of rigid-body motions SE(3) (cf. Results section). The search scheme we have introduced is capable of *uniformly* sampling the space of rigid-body rotations SO(3), where uniformity is defined according to a chosen metric. For instance, in the sampling technique from [24] we use throughout this work, uniformity involves the competing notions of *local separation* and *global coverage*. Equispaced Euler angular grids, the mainstay of all current rotationally exhaustive techniques, generate samples in SO(3) that possess neither of these desirable features. See also [29] for a more detailed consideration of sampling.
- *Reranking stage*. We have introduced the skeleton-secondary structure score (SSS), whose performance we expect to improve as the resolution of experimental cryo-EM 3D EM maps improves.
- *Optional multi-basis framework*. Our match and alignment (fitting) algorithms can use one of two popular basis expansions to perform an exhaustive search. PF^2^ *fit* —SE(3) and—SO(3) is compatible with existing FFT-based fitting schemes, while being general enough to subsume the approaches that use these schemes, approaches such as those by [3, 19, 47]. Because the NFFT is currently not as fast as the FFT, there may be situations in which the use of the FFT-based technique, regardless of its drawbacks, might be indicated. Suitable modifications of PF^2^ *fit* —SE(3) and PF^2^ *fit* —SO(3) would be applicable in these situations as well.

## Software and Data Availability

The PF^2^ *fit* software package along with a tutorial is free for academic users and available through our website: http://www.ices.utexas.edu/CVC/software/.
